# 

**Published:** 1876-10

**Authors:** 


					TO KEAJJERS AND (JORRESRONDENTiS.
I
Communications intended for publication, and exchange journals and paper;
should be addressed to the Editor of the American Journal of Dental
Science, 259 N. Eutaw St., Baltimore, Md. All letters relating to the business
of.the iournal, or containing cash remittances, to be directed to Messrs. Snowd
& Oowman. Articles for publication shoiild be received by the editor at least I
three weeks previous to the first day of the month on which the number in whic
it is designed for them to appear, is issued.
J^irThe American Journal of Dental Science will contain fiftj pages of
reading matter in each number, making a volume of not less than
Six Hundred pages
EXCLUSIVE OF ADVERTISEMENTS
T H K
AMERICAN JOURNAL
or
DENTAL SCIENCE.
F. J. S. GOKGAS, M.D.,D.D.S., Editor.
The Journal is issued on the first.of each month and contains not less
tnan Jitty pages 01 reading matter m each number, exclusive oi adver-
tisements, making a volume of six hundred pages per annum.
In each number will be found Original Communications, Corres-
pondence, Selected Articles, Monthly Summary, Bibliographical No-
tices and Editorial Matter, and every enort will be exerted to render
the Journal worthy of the patronage of the Dental profession.
A generous co-operation is respectfully solicited from' the members
of the profession ; and as the Journal has now a printing office of its
own, which materially lessens the cost ol its publication, it will be
issued at the reduced subscription price 01
$2.00 Per AnTium in Advance,
Communications are respectfully solicited on all subjects pertain-
ing to the practice of dentistry, as well as reports ot cases occurring
in dental practice.
RATES OF ADVERTISING.
1 Page.
* "
i "
1 MO.
$15 00
10 00
7 00
5 00
2 MO.
$22 50
15 00
11 00
8 00
3 MO.
$30 00
20 00
15 00
11 00
6 MO.
$50 ,00
30 00
20 00
15 00
12 MO.
$100 00
50 00
30 00
20 00
All original communications designed for publication, works for
review, new instruments ior notice, exchanges, &c.,should be directed
to the editor, 259 N. Eutaw St., Baltimore, Md.
All advertisements and subscriptions should be addressed to
SNOWDEN & COWMAN,
Publishers of the Am. Journal of Dental Science.
86 W. Fayette St. Bet. Charles & Liberty Sts.,
BALTIMORE, MD.

				

